# Secreted dual reporter assay with *Gaussia* luciferase and the red fluorescent protein mCherry

**DOI:** 10.1371/journal.pone.0189403

**Published:** 2017-12-08

**Authors:** Diana Wider, Didier Picard

**Affiliations:** Département de Biologie Cellulaire, Université de Genève, Sciences III, Genève, Switzerland; International University of Health and Welfare School of Medicine, JAPAN

## Abstract

The availability of a wide range of reporter proteins, which can easily be quantitated, has had a major impact on many fields of biomedical research. In some experiments with tissue culture cells, it is necessary to control for differences in transfection efficiency and in other expression parameters. This requirement has been very conveniently met with the popular dual luciferase assay. Its disadvantages are the requirement for cell lysis, the inability to analyze the same cells repeatedly, and the cost, at least in its most commonly used commercial format. Here we describe a novel dual reporter assay with the naturally secreted luciferase from *Gaussia princeps* as the main reporter protein and a secreted version of the red fluorescent protein mCherry as internal standard. After first measuring mCherry fluorescence in the medium, an enzyme buffer with coelenterazine as substrate is added to the same sample to trigger a glow-type luminescence of the luciferase. The simple and cheap assay can easily be adapted to a variety of experimental situations. As a case in point, we have developed a panel of *Gaussia* luciferase reporter genes for transcriptional activation assays with estrogen and glucocorticoid response elements, and with response elements for fusion proteins with the Gal4 DNA binding domain for use in mammalian cells. Our secreted dual reporter assay should be an attractive alternative to the currently available commercial kits.

## Introduction

The development of reporter genes has enormously facilitated the analysis of many steps of gene expression, from signal transduction to gene regulation to translation and protein trafficking and targeting. Chloramphenicol acetyltransferase, known as CAT, was one of the first to be introduced as a reporter enzyme for transcriptional assays in tissue culture cells [1]. Soon thereafter, the luciferase of the firefly *Photinus pyralis* was cloned and became a serious competitor for CAT because of the simplicity of the assay [2–5]. By the early 1990s, there were a whole panel of different enzymatic, including bioluminescent, and chemiluminescent reporter proteins [6,7]. In those days, although firefly luciferase was an extremely convenient reporter protein, it was not trivial to standardize its activities in transfection experiments with tissue culture cells. Typically, this involved measuring a second reporter protein in a totally different and separate assay. The introduction of the dual luciferase assay in mid-1990 was a major breakthrough [8,9]. In this assay, two enzymatically different luciferases are coexpressed and their distinct luminescent actitivies are measured sequentially after cell lysis in a relatively simple single-tube format. This reporter gene assay took many fields by storm, including the nuclear receptor field starting in the late 1990s (see for example in ref. [10]).

In its conveniently packaged commercial format, the dual luciferase assay is expensive. Non-commercial and much cheaper versions have been described [11,12], but appear not to have attracted many users yet. A common limitation that all of the protocols share is the requirement for cell lysis to measure the enzymatic activities of the two luciferases most efficiently or with a standard luminometer. This requirement obviously also precludes measuring reporter gene activities repeatedly from the same cells over time or subjected to additional treatments. To overcome this limitation, commercial and non-commercial alternatives with secreted reporter proteins such as secreted alkaline phosphatase (known as SEAP) and naturally secreted luciferases from a variety of marine species have been developed [13–17]. A notable dual reporter system combines two secreted luciferases with different substrates and enzymatic properties [15].

In many fields of research, dual reporter systems are an essential and very commonly used tool. Since none of the already existing ones are both cheap and simple, we set out to explore new options using secreted reporter proteins that have become available in the last few years. In the end, we settled on the combination of the naturally secreted luciferase from *Gaussia princeps* [18,16] and the red fluorescent protein mCherry [19], artificially induced to be secreted by fusion to a signal sequence [20,21]. We describe the basic features of this novel dual reporter system and demonstrate its application to the transcriptional regulation of reporter genes.

## Results and discussion

### Identifying the right combination of two reporter proteins

The well-known dual luciferase assay consists of the consecutive enzymatic analysis of the activities of the *Photinus pyralis* ("firefly") and *Renilla reniformis* ("Renilla") intracellular luciferases. This inspired us to consider initially only luciferases to set up a new dual reporter system. Our efforts were directed at mammalian tissue culture cells, but the system should be applicable to tissue culture cells of other species as well. We set the following criteria: (i) Both enzymes or reporter proteins should be secreted; (ii) the assays should be sensitive, simple, and cheap; (iii) the assays should ideally be doable in a single-tube (or well) format either simultaneously or consecutively; (iv) the assay should require at most a standard laboratory fluorometer and/or luminometer. Although there are by now a whole range of luciferases with different biochemical and optical characteristics [17,22], it quickly became apparent that no combination would satisfy all criteria. Only few luciferases are naturally secreted, and as substrates they all either use coelenterazine or a coelenterazine derivative, or the highly unstable luciferin variant vargulin. Although the *Gaussia princeps* luciferase (Gluc) [18,16], for which coelenterazine is the substrate, appeared to be a good choice, we still needed a second reporter with different enzymatic characteristics. Indeed, we had a preference for an enzyme over a fluorescent protein because of the inherent signal amplification. However, despite an earlier report describing the targeting of the standard firefly luciferase to the cell surface to measure extracellular ATP [23], our own attempts to getting this intracellular luciferase to be efficiently secreted into the medium in a functional form proved to be unsuccessful. Even mutating some of the cysteine residues, known to be dispensable for enzymatic activity [24], did not help. Whatever the reasons for this failure are, we turned to secreted fluorescent proteins as alternatives. Yang and colleagues had just demonstrated that both EGFP and mCherry could be secreted by fusion to a secreted protein or signal sequence [20,21]. Thus, we decided to keep Gluc for its exquisite sensitivity and high dynamic range and to combine it with secreted mCherry as internal standard.

**Fig 1:**
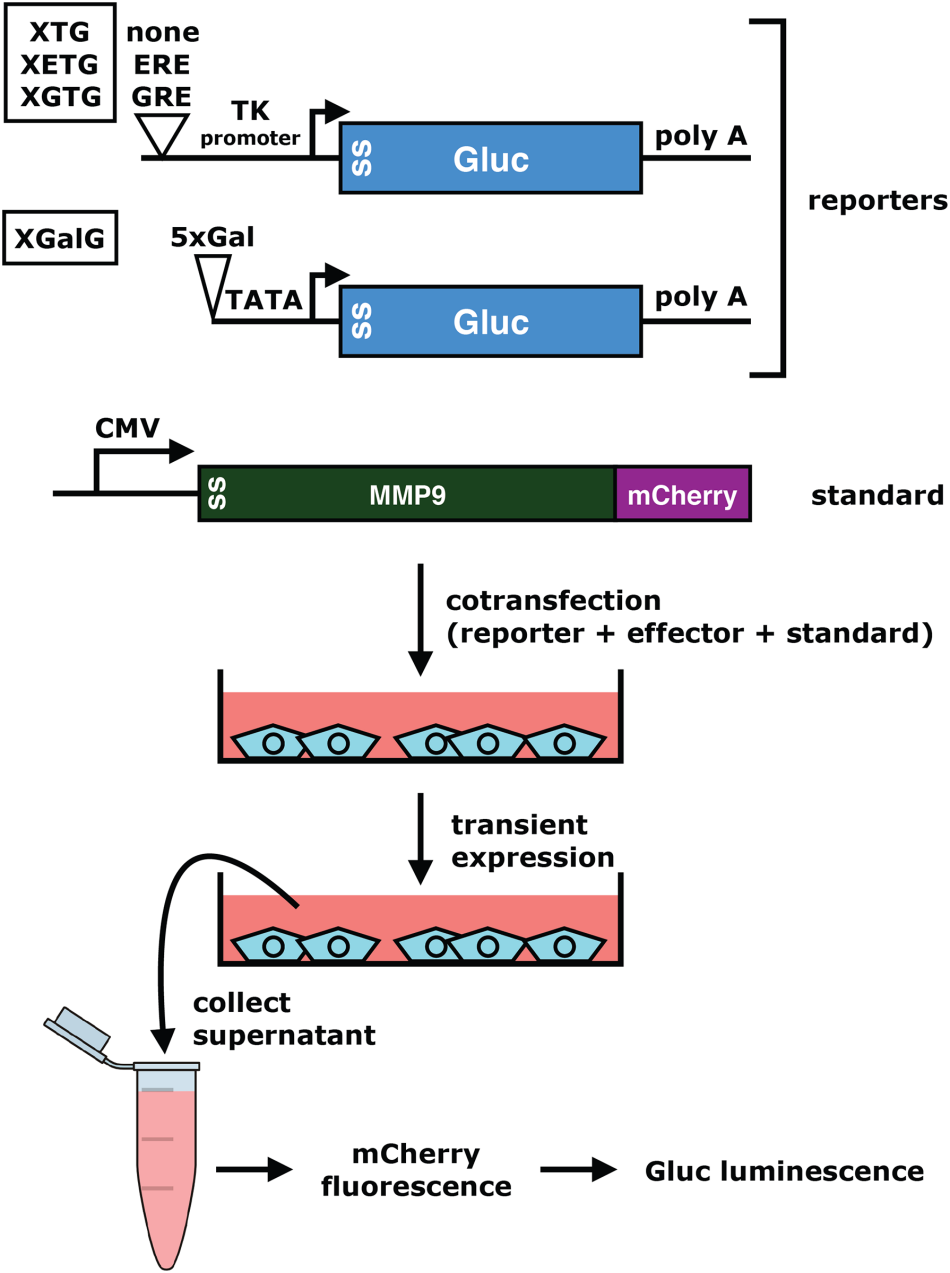
General scheme of the secreted dual reporter assay. The strategy applies to any combination of Gluc reporter and mCherry internal standard. The structures of the specific reporter plasmids used here are indicated with their names in rectangles on the left. The sizes of the different elements are not to scale. SS, signal sequence; TK, thymidine kinase; 5xGal, 5 copies of Gal4 binding site.

Fig 1 illustrates the general setup that we have established. Gluc is used as the reporter protein, for example encoded by genes reporting on transcriptional regulation. Gluc has its own signal sequence of 17 amino acids, and it is therefore secreted through the canonical pathway. A fusion protein of human matrix metallopeptidase 9 (MMP9) to mCherry ("mCherry" for short) [20,21] is used as internal standard. mCherry fluorescence corrects for variations in numbers of cells, transfection and expression efficiency as well as effects of experimental treatments on the secretory pathway. Upon expression in tissue culture cells, mCherry and Gluc accumulate in the cellular medium. Their fluorescent and enzymatic activities, respectively, can then easily be measured in collected medium. Typically, this is done by transferring aliquots of the medium to multiwell dishes. After measuring the mCherry fluorescence, an appropriate reaction buffer is added to the same wells to record the Gluc luminescence. The data are processed by standardizing the Gluc enzymatic activity to the mCherry fluorescence for each sample.

Several additional technical details must be mentioned at this point: (i) Coelenterazine displays some autoluminescence background because of autooxidation [25]; this would adversely affect the sensitivity of the Gluc assay, but can be prevented by including some sodium iodide (NaI) in the assay buffer (according to US patent 08512968). (ii) The Gluc in all of our constructs is the M43I point mutant (numbering is from first amino acid of mature protein). M43I, in contrast to wild-type Gluc, yields a glow-type luminescence, which is further stabilized by including 0.1% Triton X-100 in the reaction buffer [26]. (iii) Although a wide range of media and media supplements are compatible with the assay, some display autofluorescence, which can contribute a significant background to the mCherry fluorescence; we therefore adopted the use of Opti-MEM after transfection as previously suggested [21]. (iv) In its current version, our internal standard is a full-length MMP9 fusion to mCherry; it is conceivable that it retains the enzymatic activity of its MMP9 moiety, which might be relevant in some experimental situations.

### Basic features of the dual reporter assay

**Fig 2:**
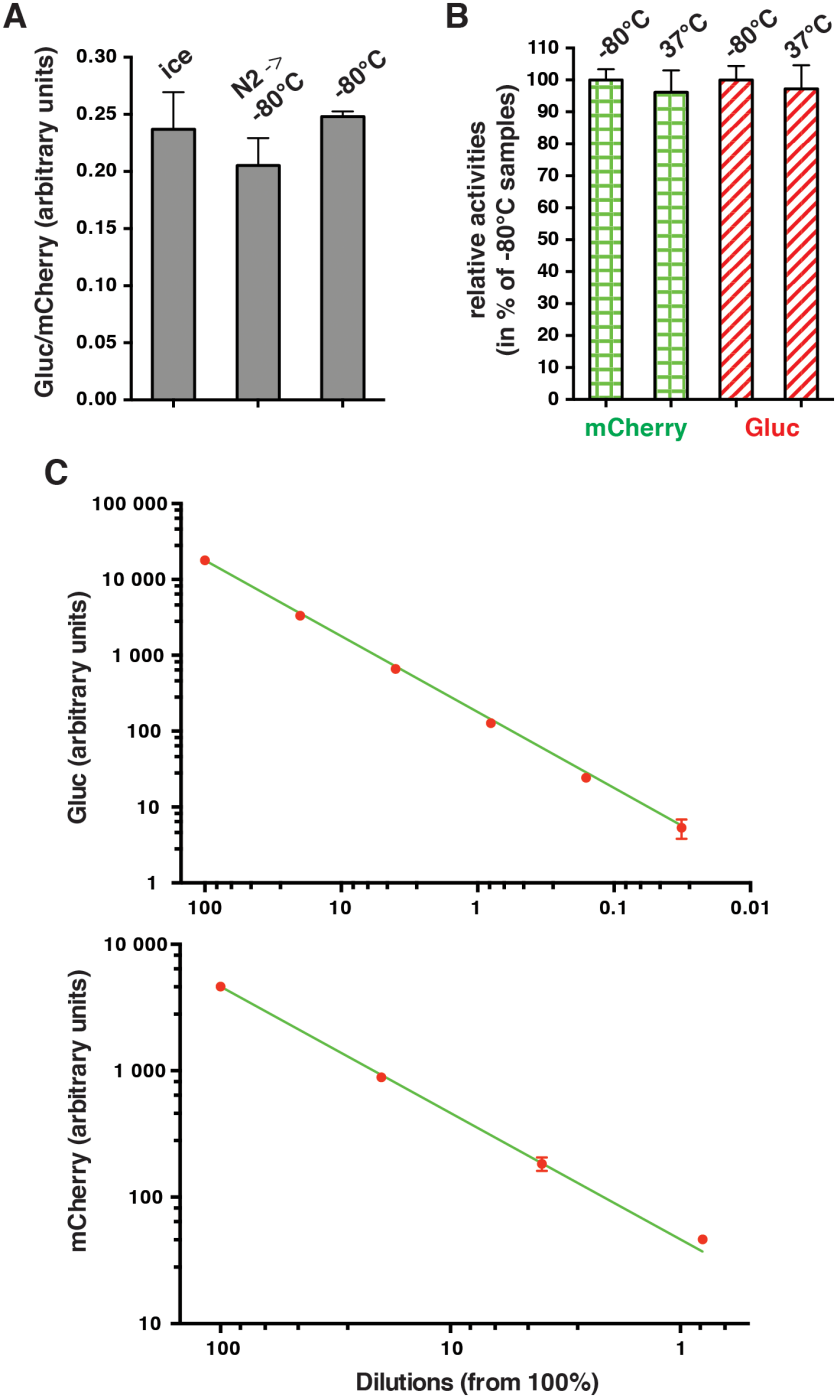
Characteristics of the secreted reporter proteins in tissue culture medium. (**A**) Freezing tests. Supernatants of HeLa cells cotransfected with XETG and mCherry were placed on ice and then aliquots were flash-frozen or not in liquid nitrogen before transferring to -80°C; Gluc and mCherry activities of all aliquots were measured in parallel 45 minutes later. Individual bars represent the average of triplicate samples and error bars show the standard deviation. Apparent differences are not statistically significant (p > 0.2). (**B**) Stability tests of Gluc and mCherry in medium. Supernatants of HCT116 cells cotransfected with XETG and mCherry were collected 40 hours after transfection with PEI and either kept frozen at -80°C or in a tissue culture incubator at 37°C in a fresh plate without cells for another 24 hours. Individual bars represent the average of triplicate samples and error bars show the standard deviation. Activities of the frozen aliquots were set to 100%. Apparent differences are not statistically significant. (**C**) Activities in serial dilutions. The double-logarithmic graphs show the results of triplicate samples (red dots) with error bars indicating the standard deviation in comparison to the theoretical values of a linear stepwise dilution (green lines); the respective average of the undiluted samples were set to 100%. For mCherry (bottom panel), the samples were the same as for the freezing tests, and they were diluted with Opti-MEM in steps of 5; for Gluc (top panel), supernatants were from HeLa cells cotransfected with XGalG and Gal4.VP16, and they were complemented with 10% fetal calf serum (FCS) before dilution in steps of 5 with Opti-MEM complemented with 10% FCS. Apparent deviations from the predicted values are not statistically significant (p > 0.2).

We determined whether supernatants can be frozen for storage and whether they can be diluted. We tested the possibility to freeze supernatants by placing them at –80°C with and without prior flash-freezing in liquid nitrogen. As can be seen in Fig 2A, aliquots of supernatants can be directly placed at -80°C for long-term storage without loss of activity. The apparent reduction upon flash-freezing first in liquid nitrogen is not statistically significant. We have not directly tested whether samples can be stored for several weeks or months, but these results demonstrate that the freeze/thaw process *per se* does not reduce the activity of the reporter proteins. Another important parameter is whether mCherry and Gluc are differentially stable in medium under growth conditions as this might compromise the faithfulness of the assay. We tested this directly with medium from transfected cells. At the 40 hours time point (see below), we set aside an aliquot at -80°C and left another in the 37°C tissue culture incubator in a new plate without cells for an additional 24 hours. As Fig 2B shows, there is not statistically significant difference between the two samples indicating that the two proteins are basically stable once secreted into the medium. A serial dilution over several orders of magnitude yields the expected values (Fig 2C). It should be pointed out that 10% serum must be added to preserve Gluc activity in diluted supernatants and that this is in turn not compatible with fluorescence measurements since it contributes a substantial background. Hence, dilutions must be done with and without serum for Gluc and mCherry, respectively. Nevertheless, these results suggest that large fold-differences can be faithfully reported by the assay (see also below), and that activities beyond the range of a particular instrument could still be measured by diluting the supernatants before the measurement.

**Fig 3:**
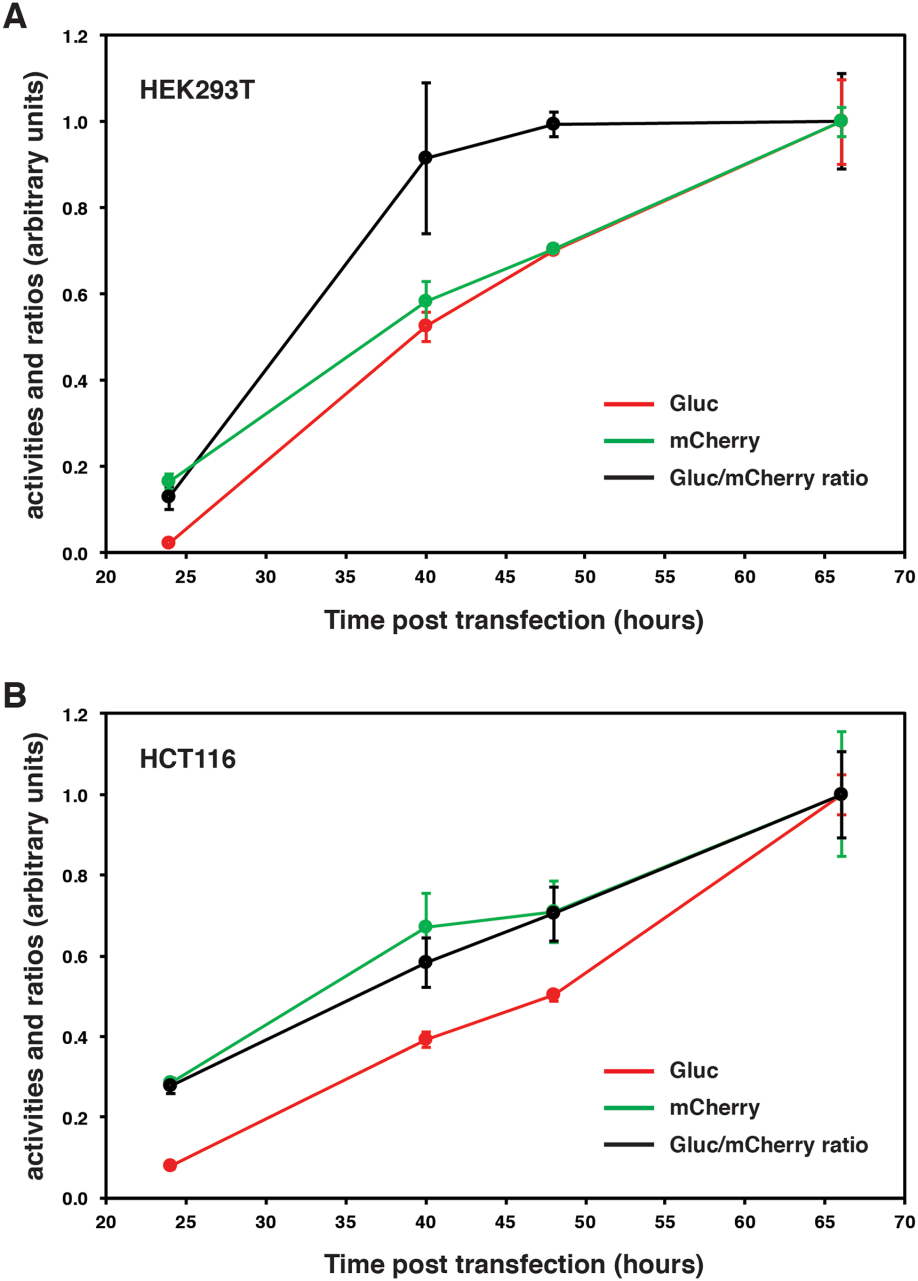
Time-course assay. (**A**) Time-course experiment with transfected variant HEK 293T cells. They were cotransfected with equal amounts of the plasmids XETG and mCherry with the calcium-phosphate coprecipitation technique. Individual data points represent the average of triplicate samples (except for the 48 hour time point, which is the average of duplicate samples) and error bars show the standard deviation. All values were standardized to those of the 66 hour time point (arbitrarily set to 1.0). (**B**) Time-course experiment with transfected HCT116 cells. Exactly the same as in panel **A** except that cells were transfected with PEI and that all samples are in triplicates.

The standard time-course for expression of transiently transfected plasmids has been known for over 35 years; for human HeLa cells, the maximum accumulation of an exogenously expressed intracellular protein was shown to be reached about 48 hours post-transfection [27]. Since our dual reporter assay requires both protein expression and secretion into the medium, the time-course might be slightly different. We found that the fluorescence activity of mCherry accumulating in the medium has a slightly faster time-course than the Gluc activity both in human embryonic kidney cells (HEK 293T) and in HCT116 colon cancer cells (Fig 3). Both proteins continue to accumulate over at least 66 hours, and their ratio stabilizes at about 48 hours with HEK 293T, but not HCT116 cells. The slightly different kinetics observed with the two cell lines could be due to cell-type differences or to the fact that HEK 293T and HCT116 cells were transfected with different reagents (calcium-phosphate and polyethylenimine (PEI), respectively). Intracellular reporter proteins decline beyond 48–60 hours [27], most likely because the transfected expression plasmids begin to be shut down and proteins turn over. In contrast, secreted reporter proteins may in part escape the intracellular turnover and continue to accumulate for some time. Thus, depending on the specific experimental question, the time point can be exploited as an additional experimental parameter. For transcriptional assays, it may be best to stick to an earlier time point such as 42 hours to ensure that the secreted proteins report on events at the peak of transient intracellular expression.

### Application to transcriptional activation assays

**Fig 4:**
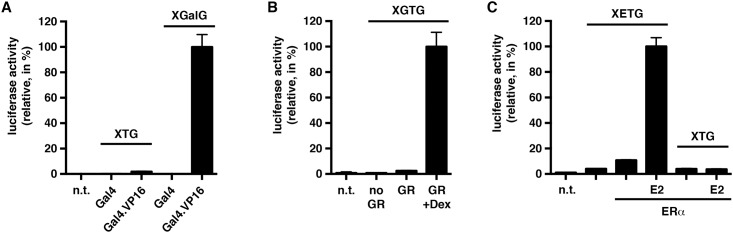
Transcriptional activation assays. The secreted dual reporter assay was used for three reporter systems tested in HeLa cells. Gluc luciferase activities were standardized to mCherry and calculated as % of the maximal activation of each reporter/effector pair set to 100%. Bars represent averages of triplicate samples and error bars the standard deviation. (**A**) Transcriptional activation of the Gal4 reporter plasmid XGalG by Gal4.VP16. Gal4, the DNA binding domain of Gal4; n.t., medium from non-transfected cells. (**B**) Transcriptional activation of the GR reporter plasmid XGTG by GR in the presence of 100 nM Dex. "no GR", cells cotransfected with empty expression vector. (**C**) Transcriptional activation of the ERα reporter plasmid XETG by ERα in the presence of 100 nM E2.

Dual luciferase assays have been immensely popular to assess the transcriptional activities of transcriptional regulatory sequences and the factors that regulate them. The nuclear receptor field promptly adopted this type of assay when it became available in the late 1990s (see for example in ref. [10]). We decided to generate a panel of standard Gluc reporter genes for mammalian cells. The basic reporter plasmid for several of them is recombinant XTG, and it has the following features: X stands for a plasmid backbone lacking the cryptic AP-I site of many commonly used cloning vectors [28]; T stands for the promoter region of the *Herpes simplex* virus thymidine kinase gene (nucleotides -109 to +52); G for Gluc. The "core" of XGalG is similar except that it only has a minimal promoter with a TATA box. In XGalG, XGTG and XETG, response elements for Gal4, the glucocorticoid receptor (GR) and estrogen receptor α (ERα), respectively, are inserted upstream of the promoter (Fig 1). The detailed maps and sequences are available as Supporting Information (S1 Maps sequences). The reporter plasmids were all tested in HeLa cells by cotransfection with expression vectors for the appropriate effectors (Fig 1). Gluc expression of the Gal4 reporter plasmid XGalG is strongly induced by the potent synthetic activator Gal4.VP16 consisting of the Gal4 DNA binding domain fused to the activator domain VP16 (Fig 4A). Similarly, XGTG is switched on by GR in the presence of the synthetic glucocorticoid dexamethasone (Dex) (Fig 4B), and XETG by ERα in the presence of the physiological estrogen 17β-estradiol (E2) (Fig 4C). Note that the reporter plasmid XGTG should also work with the mineralocorticoid, progesterone and androgen receptors because of overlapping DNA binding specificities. We conclude that these reporter plasmids constitute a useful new set of tools, which could replace the corresponding intracellular luciferase reporters and *Renilla* internal transfection standard for these types of assays.

### Conclusions

20 years ago, when the intracellular dual luciferase was introduced, it constituted a huge step forward compared to the reporter enzymes and assays that had previously been available. It is not surprising that it very rapidly conquered the world of transcriptional activation assays and beyond. Its convenience, sensitivity and the fact that it could be adapted to high throughput analyses constituted important selling points. Commercial kits, notably of one brand, are of a quality and reliability that are hard to match with home-made versions. There are commercial alternatives to the popular combination of luciferases, including of secreted reporter proteins. An example of that is the combination of Gluc with SEAP, but this involves two separate reactions. Using only fluorescent reporters may be appropriate in some instances [20,21], but in others, the amplification of at least one of the signals by an enzymatic reaction may be essential. Our "secreted dual reporter assay" constitutes a powerful non-commercial alternative for many applications (Table 1). Since the reporter proteins are secreted into the medium, no cell lysis is required and repeated assays of the same cells become feasible. For example, the medium could be collected at some point, the same cells could be washed, subjected to a particular treatment and reanalyzed again a few hours later. Thus, with this new assay, more complex experimental protocols become conceivable.

**Table 1:** Comparison of secreted and intracellular dual reporter assays.

	Secreted dual reporter assay (Gluc & mCherry)	Dual luciferase assay ^1^ (firefly & Renilla)	Dual luminescence assay ^2^ (Gluc & SEAP)	Secreted dual luciferase assay ^3^ (Gluc & Cypridina)
Cell lysis	not required	required	not required	not required
Special lysis buffer	N.A. ^4^	yes	N.A.	N.A.
Internal transfection standard	yes	yes	yes	yes
Enzymatic reactions	one	two	two	two
Single tube/well assay	possible	possible	no	possible
Number of measurements needed per sample	two	two	two	two
Repeated measurements of same live samples	possible	no	possible	possible
Required reaction mixes	one	two	≥ two	two
Required instrument	fluorometer/luminometer	luminometer	luminometer	luminometer
HTP possibility	yes	yes	yes	yes
Price per sample ^5^	9 cents	≥ 2.47 $	≥ 1.20 $	≥ 0.50 $

^1^ Kits as commercialized for example by Promega; for non-commercial options, see text.

^2^ Kits as commercialized for example by GeneCopoeia or TaKaRa.

^3^ Separate kits are sold by a number of suppliers, including kits for *Cypridina* luciferase for example by NEB, Targeting Systems, and Thermofisher; note that the non-commercial option for this dual luciferase assay mentioned before [15] requires the non-standard luciferin vargulin, which is cheap but highly unstable in solution.

^4^ Abbreviations: N.A., not applicable; HTP, high throughput.

^5^ Price calculations: specific products required for the final assays assuming cells are transfected in a 6-well plate; the use of Opti-MEM accounts for about 90% of the price in the case of the secreted dual reporter assay; for the dual luciferase assay, the catalog price of the Promega kit for 1'000 samples was used to calculate the price per sample in Switzerland, but the prices in Swiss francs were converted to US$ (June 2017).

## Materials and methods

### Reagents

Native coelenterazine was from BIOSYNTH, "Opti-MEM I Reduced Serum Medium, without Phenol Red" (Opti-MEM) from Life Technologies. 96-well dishes (black flat bottom, non-binding surface polysterene) for fluorometry and luminometry were from Corning. Gluc buffer (90 mM Tris-HCl pH 8.0, 15 mM NaCl, 0.3% Triton X-100, 75 mM NaI, 10 μM native coelenterazine) was prepared fresh from stocks at room temperature and protected from light; coelenterazine was added just before use from a 5 mM stock in acidified methanol (stored at -80°C).

### Plasmids

The MMP9-mCherry internal standard is expressed under the control of the CMV enhancer/promoter from expression vector pCI-neo and has been described [21]. The parent plasmid for Gluc reporters was recombinant pTK-GLuc from New England Biolabs. The mutagenesis to the M43I version pTK-GLuc(M43I) was done by Mutagenex. Appropriate DNA fragments of plasmids XTL, XETL, and XG46TL [29] were combined with portions of pTK-GLuc(M43I) in cloning vector BLUESCRIPT M13+ to generate XTG, XETG, and XGTG, respectively. To construct XGalG, a fragment containing 5 Gal4 binding sites and a minimal promoter were excised from reporter plasmid GK1 [30] and combined with appropriate fragments from pTK-GLuc(M43I) in the cloning vector pSP73. The expression plasmids HEG0 [31], pC7G [29], and pSCTEV-gal93 [32] were cotransfected to express ERα, GR, and the Gal4 DNA binding domain (amino acids 1–93), respectively. Plasmid GAL93.VP16 for expression of Gal4.VP16 was constructed by inserting the coding sequences of VP16 into pSCTEV-gal93. Maps and sequences of plasmids XTG, XETG, XGTG, and XGalG are available in Supporting Information (S1 Maps sequences).

### Cell culture and transfection

Standard and commonly used established human cell lines were used. In some cases, they were obtained from ATCC. Note that for the purposes of these experiments, their exact background and/or genetic makeup are irrelevant. Cell lines included HeLa cells, a more adherent variant of HEK 293T cells, and human HCT116 colon cancer cells, which were all cultured in DMEM complemented with 10% FBS. For transcriptional activation assays with steroid receptors, cells were switched to DMEM without phenol red containing 10% charcoal-stripped FBS at least one day prior to transfection. Cells were transfected with the calcium-phosphate coprecipitation technique or with PEI MAX (from Polysciences) with 5 μg total DNA per well of a 6-well dish. For transcriptional activation assays, the standard mix per well was 2.5 μg reporter plasmid, 200 ng mCherry, 2.3 μg effector expression vector. The day after transfection, the medium was changed to Opti-MEM.

### Secreted dual reporter assay

Supernatants (medium) were collected 42 hours after transfection unless indicated otherwise. Floating cells and debris were removed by a quick spin. 100 μl were transferred into a well of a black 96-well dish. mCherry fluorescence was determined first by exciting at 570 nm and measuring emission at 610 nm with a 100 msec time delay. Gluc luminescence was then triggered by adding 50 μl Gluc buffer per well. Both fluorescence and luminescence were measured with a Biotek Cytation 3 multiwell reader. A detailed step-by-step protocol is available at http://dx.doi.org/10.17504/protocols.io.kbwcspe [PROTOCOL DOI].

## Supporting information

S1 Maps sequences Maps and sequences of the reporter plasmids. A zipped archive with pdf and xdna files with the maps and sequences, respectively, of plasmids XTG, XETG, XGTG, and XGalG.
(ZIP)
