# Supplementary figures and images for: Secreted dual reporter assay with Gaussia luciferase and the red fluorescent protein mCherry
Source: PLoS One. 2017 Dec 8;12(12):e0189403. doi: 10.1371/journal.pone.0189403 (PMC5722324; doi:10.1371/journal.pone.0189403)

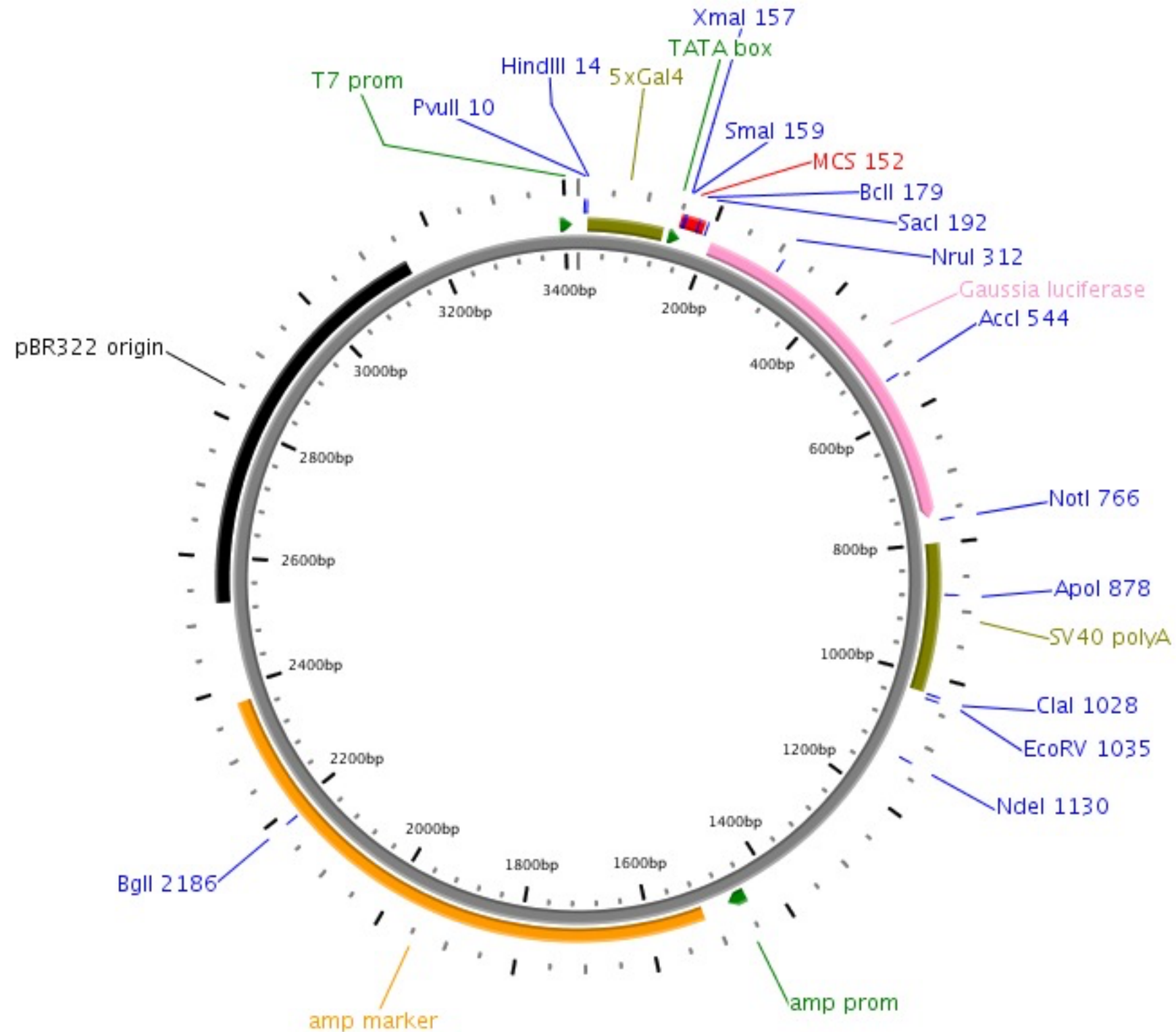

Supplement: S1 Maps sequences — A zipped archive with pdf and xdna files with the maps and sequences, respectively, of plasmids XTG, XETG, XGTG, and XGalG. (ZIP) [file pone.0189403.s001.zip › Supporting Information/XGalG.pdf]

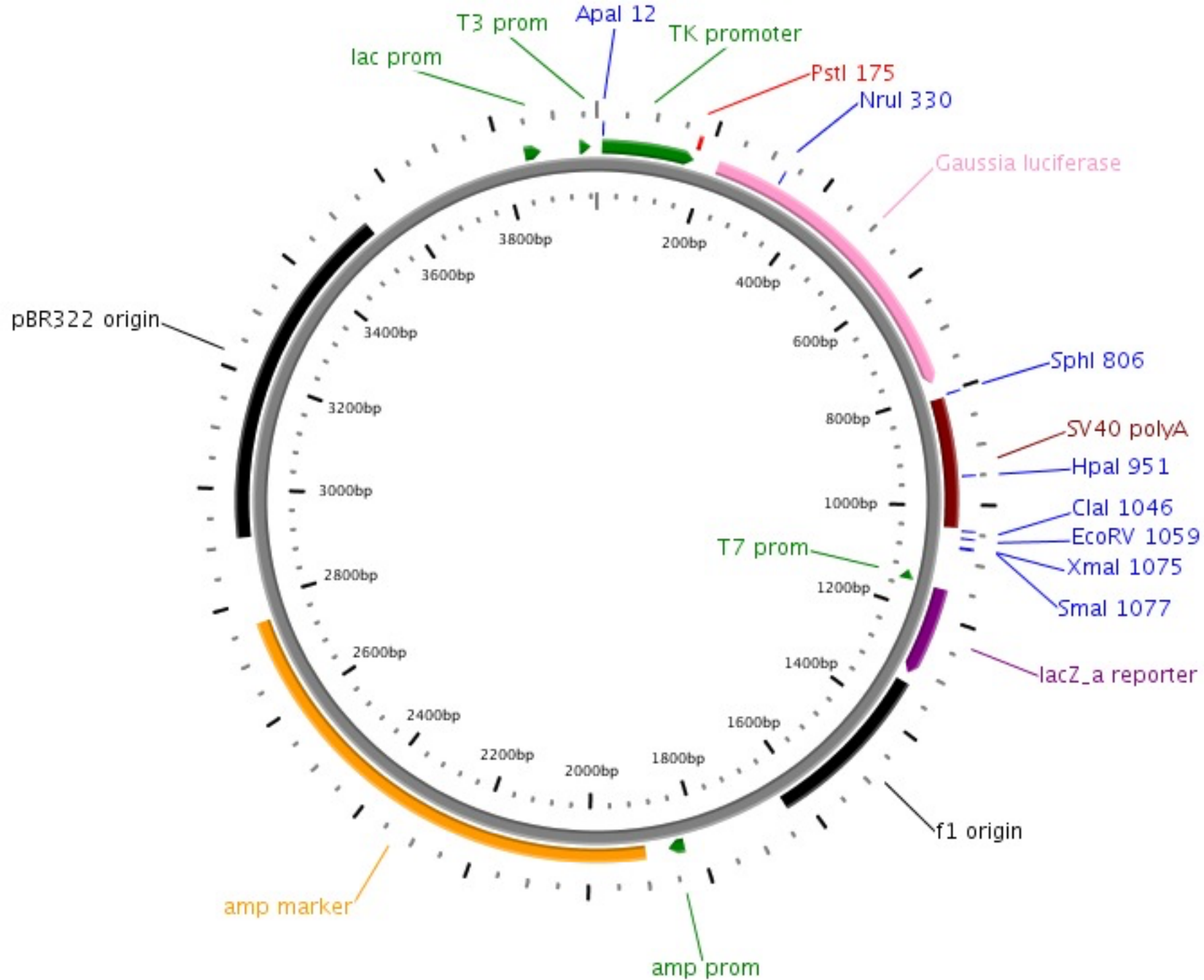

Supplement: S1 Maps sequences — A zipped archive with pdf and xdna files with the maps and sequences, respectively, of plasmids XTG, XETG, XGTG, and XGalG. (ZIP) [file pone.0189403.s001.zip › Supporting Information/XTG.pdf]

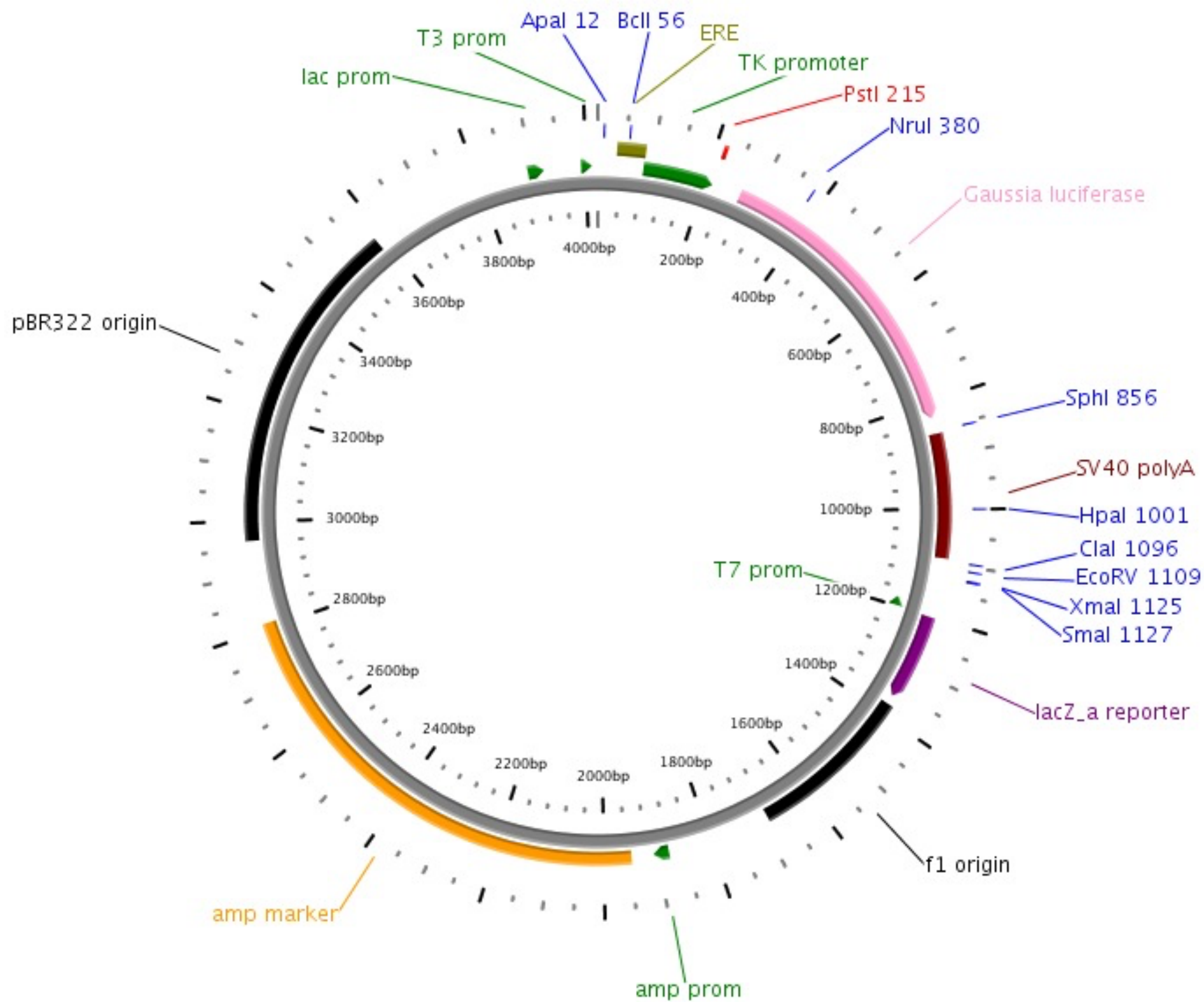

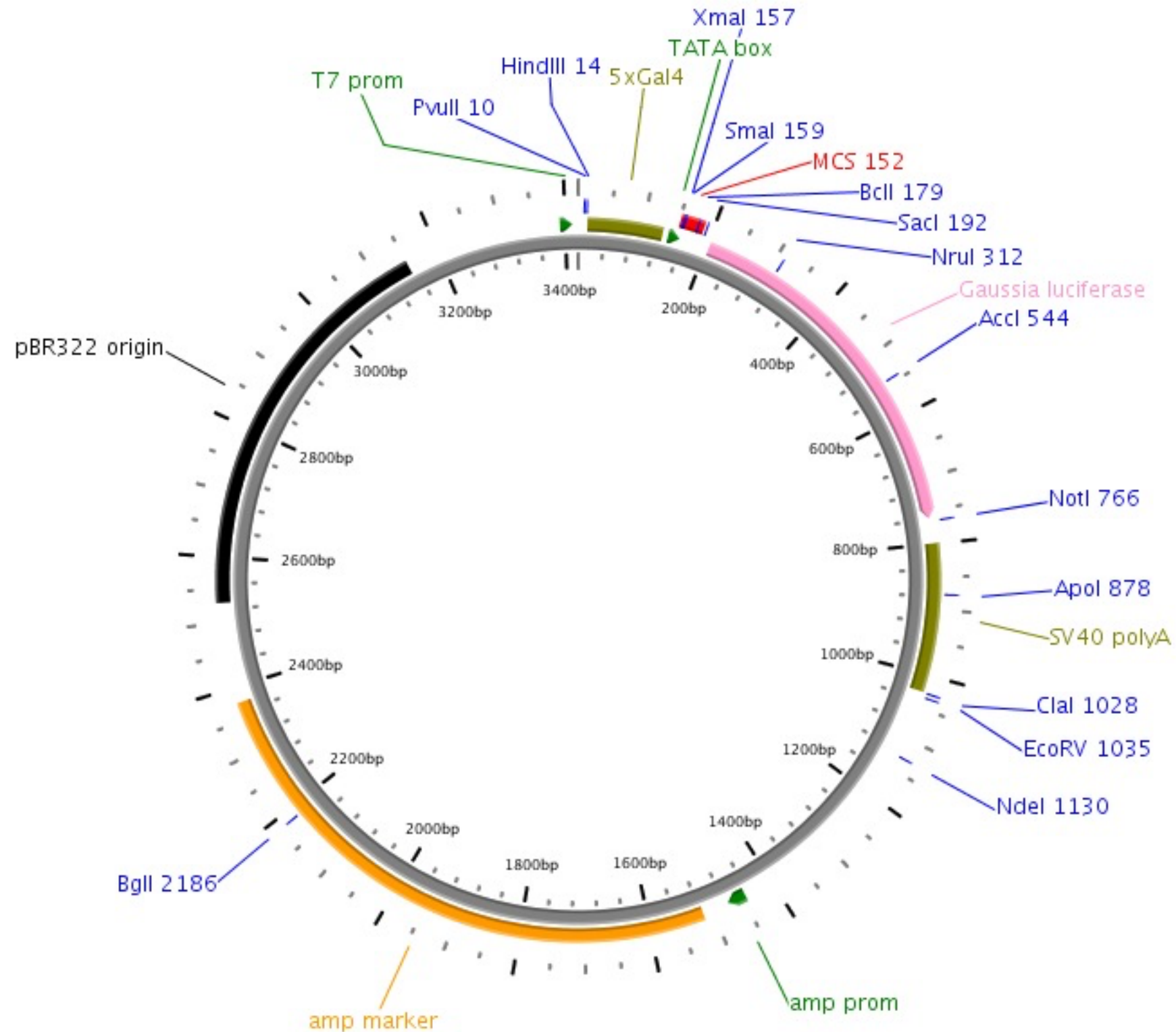

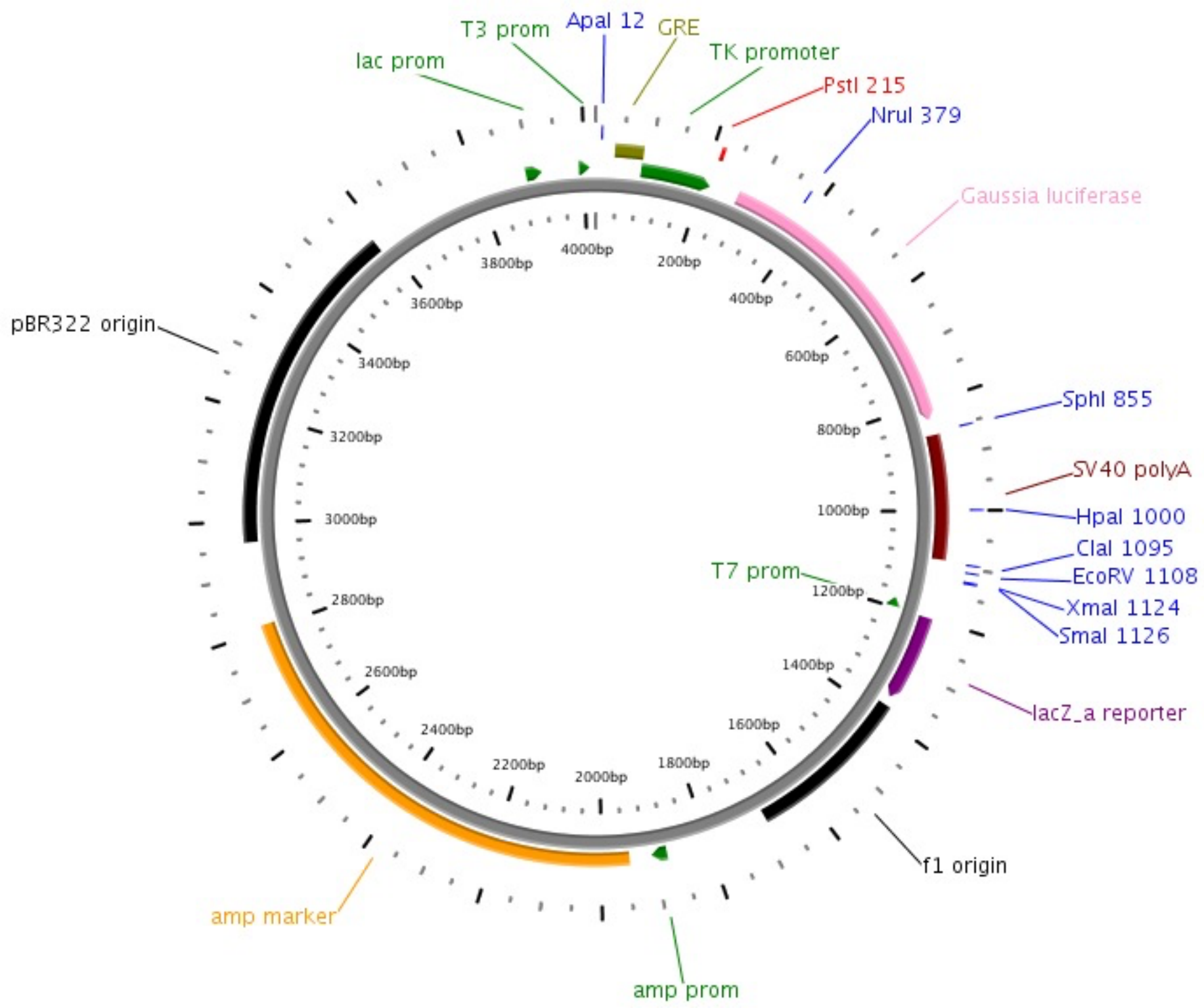

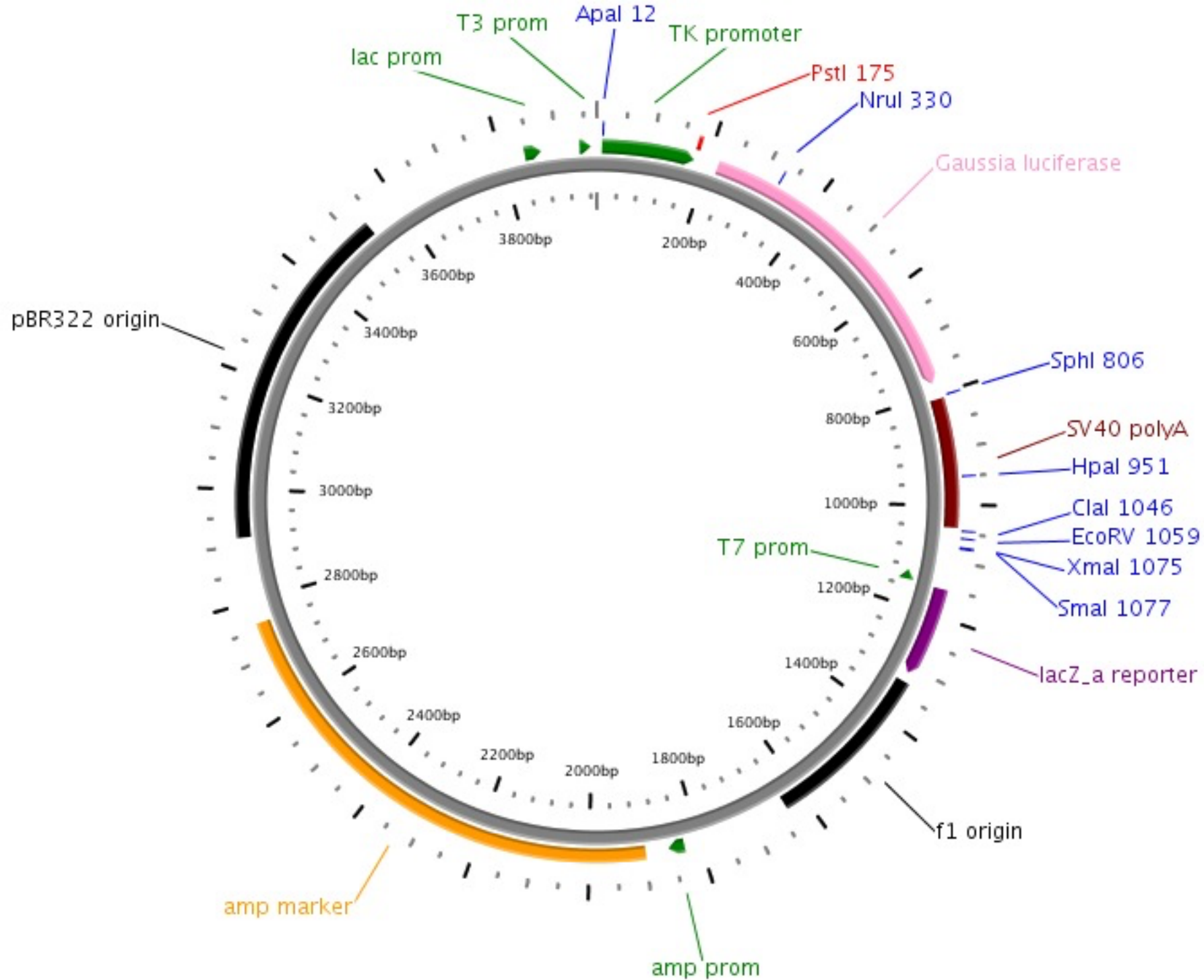

Supplement: S1 Maps sequences — A zipped archive with pdf and xdna files with the maps and sequences, respectively, of plasmids XTG, XETG, XGTG, and XGalG. (ZIP) [file pone.0189403.s001.zip › Supporting Information/XETG.pdf]

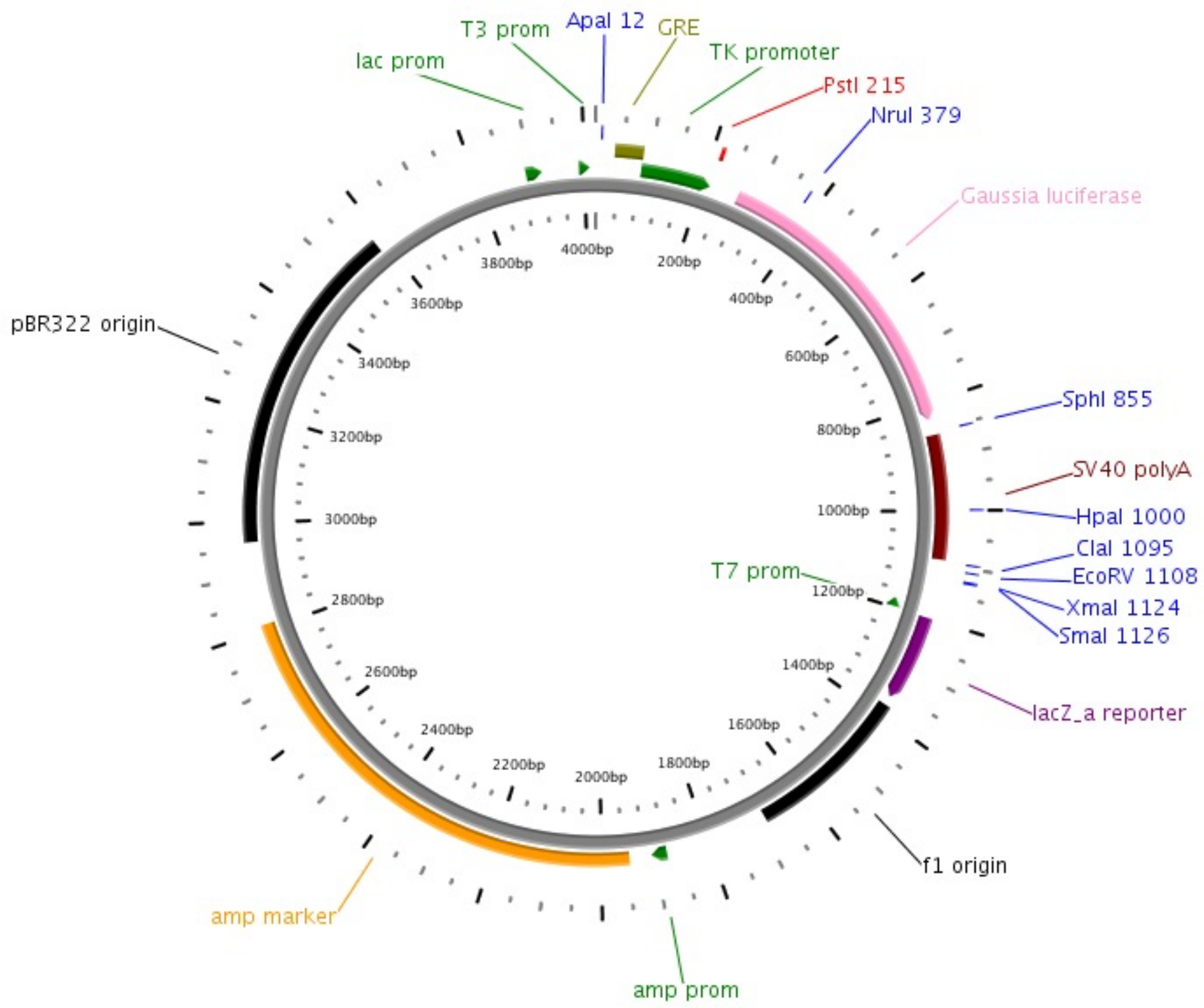

Supplement: S1 Maps sequences — A zipped archive with pdf and xdna files with the maps and sequences, respectively, of plasmids XTG, XETG, XGTG, and XGalG. (ZIP) [file pone.0189403.s001.zip › Supporting Information/XGTG.pdf]
